# Adherence to plant-based dietary patterns and anthropometric indices among primary school girls in Kerman: A cross-sectional study

**DOI:** 10.1371/journal.pone.0298454

**Published:** 2024-02-23

**Authors:** Nooshin Jannati, Mohammad Reza Mahmoodi, Leila Azadbakht

**Affiliations:** 1 Department of Community Nutrition, School of Nutritional Sciences and Dietetics, Tehran University of Medical Sciences (TUMS), Tehran, Iran; 2 Faculty of Public Health, Institute of Neuropharmacology & Department of Nutrition, Physiology Research Center, Kerman University of Medical Sciences, Kerman, Iran; The University of Texas at El Paso, UNITED STATES

## Abstract

The objective of the study was to evaluate the association between adherence to plant-based dietary patterns and anthropometric indices among primary school girls in Kerman. This cross-sectional study included 330 girls aged 6–12. A reliable and validated dish-based food frequency questionnaire was used to collect dietary data. Weight, height, and mid-arm circumference were measured, and z-score charts from the World Health Organization for girls between the ages of 5 and 19 were utilized. We used Satija et al. method to calculate plant-based diet index scores. After adjusting for age and energy, participants in the higher tertile of the overall plant-based diet index (PDI) had a lower height-for-age z score (HAZ) (0.38±0.12 vs. 0.59±0.12 kg; P = 0.033). Higher unhealthful plant-based diet index (uPDI) scores were significantly associated with decreased HAZ in models 2 (p = 0.028) and 3 (p = 0.035). A higher PDI score was associated with lower odds of being underweight and overweight, respectively (Model 1: P trend = 0.007, <0.001; Model 2: P trend = 0.010, 0.001). A significant inverse association was found between healthful plant-based diet index (hPDI) scores and underweight risk in the crude and adjusted models. (Model 1: P trend = 0.021; model 2: P trend = 0.018; Model 3: P trend = 0.031). Higher uPDI scores were associated with increased odds of being overweight and obese in all three models (Model 1: p trend<0.001; Model 2: p trend<0.001; Model 3: p trend = 0.001). We concluded that children who followed a plant-based diet had lower odds of being overweight and obese. Higher scores on the hPDI were linked to a decreased risk of being underweight, while higher scores on the uPDI were associated with an increased risk of being overweight and obese. The study suggests healthy plant-based diet may benefit children’s weight and growth.

## Introduction

Child growth assessment is critical for health promotion and human development [1]. Anthropometric measurement is a reliable method to assess children’s nutritional status and growth [2]. The prevalence of overweight, wasting, and stunting is 5.6%, 6.9%, and 21.3% globally [3,4].

In Asia, weight abnormalities rates during childhood and adolescence, including underweight, overweight, and obese, are high [5,6]. The prevalence of obesity, overweight, and underweight among students in Kerman were 9%, 10%, and 17% respectively [7]. Overweight or obese schoolchildren are at risk of chronic diseases such as high blood pressure and type 2 diabetes [8]. On the other hand, child thinness can lead to delayed maturation, poor muscle strength, and later performance [9]. Malnutrition during child growth can cause stunting, which increases the risk of nutrition-related chronic diseases in adulthood [10].

Various factors influence child growth. Diet is one of the most important factors during a child’s development [11]. Studies have shown that a plant-based diet, characterized by a significant consumption of plant foods and limited consumption of animal foods, reduces weight and cardiovascular risk factors in children [12]. Although plant foods such as fruits and vegetables are healthy, some plant foods contain a significant amount of sugar, refined carbohydrate, toxins or chemicals (e.g., vegetables, rice, potatoes and refined grains) that can have negative impacts on one’s health [13–18]. Food safety is a fundamental principle in obtaining plant food sources, and nanotechnology may be one of the most important approaches to achieving adequate food safety and nutritious food effectively. However, the results from studies demonstrated that ZnONP treatment inhibits plant accumulation of toxic heavy metals, and promotes the uptake of essential micronutrients [15]. Zinc Oxide Nanoparticles (ZnO-NPs) also significantly reduced the plant uptake of heavy metals such as Nickel [16]. The results of a study showed the transformation of Plant-Derived Medicinal Compounds such as flavonoids, in response to metallic oxide nanoparticle contamination [17]. On the other hand, the co-exposure to ZnONPs and PFOA/GenX resulted in lower oxidative stress than the plants exposed to PFOA or GenX alone [18].

The hPDI focuses on high-quality plant foods that have positive effects on health outcomes. In contrast, the uPDI focuses on less nutritious plant foods that increase the risk of chronic conditions [19].

Previous studies have focused on a vegetarian diet [20–22]. However, in Middle Eastern countries, high consumption of plant-based food like white rice and bread has several health effects. Therefore, the health benefit of a plant-based diet should be considered instead of focusing on a vegetarian diet. A systematic review showed conflicting evidence on the effect of vegetarian diets on child growth [20]. A study on six years old Iranian children showed an inverse relationship between fish/white meat and underweight/wasting, and a positive association between protein intake of fish/white meat and the risk of overweight/obesity [21].

The growth and nutritional status of girls in the 6–12 age group are fundamental because they are future mothers, and the community’s health depends on their health. To the best of our knowledge, no study has evaluated the association between plant-based dietary patterns and anthropometric indices in Kerman. Considering the high rates of child malnutrition in Kerman, we aim to investigate the association between adherence to plant-based dietary patterns and selected anthropometric indices among primary school girls in Kerman.

## Materials and methods

### Study population

This cross-sectional study was conducted on 330 children aged 6–12 in Kerman in 2022. We used the following formula to calculate sample size based on the mean and standard deviation of BMI among 6-12-year-old Iranian children (Mean (± SD) = 16.0 ± 2.9 kg/m^2^) [23], with d = 2%, alpha = 0.05, and power = 80%.


$$N=[{\left(z1‐\alpha /2\right)}^{2}\times s^{2}]/d^{2}$$

The recruitment period was from 1 January 2022 to 3 March 2022. We selected cases using cluster random sampling methods. The inclusion criteria were as follows: 1) completing informed consent; 2) participants between the ages of 6 and 12; 3) lack of chronic diseases such as diabetes, congenital metabolic diseases such as maple syrup disease or phenylketonuria, thyroid disease, epilepsy, or asthma. 4) Not taking corticosteroids, thyroid drugs, diabetes drugs, epilepsy drugs, or allergy drugs. Exclusion criteria included participants whose parents did not complete the informed consent document. The study protocol was approved by the Ethical Committee of Tehran University of Medical Sciences (IR.TUMS.MEDICINE.REC.1400.603). The written consent form was completed by all guardians.

### Dietary intake assessment

Dietary intake was assessed using a dish-based food frequency questionnaire (FFQ) to evaluate participants’ dietary intake based on their consumption in the last year, categorized as daily, weekly, or monthly intake. We evaluated the validity and reliability of this FFQ and reported the results in the Validity and reliability of food frequency questionnaire section. The energy and nutrients were calculated by NUTRITIONIST IV software (First Data Bank, San Bruno, California), modified for Iranian foods measurements.

### Construction of plant-based dietary scores

Plant-based dietary scores were calculated using the method of Satija et al. [24], which involved creating 19 food groups and categorizing them into healthy plant foods (whole grains, fruits, vegetables, nuts, legumes, vegetable oils, and tea/coffee), less healthy plant foods (Refined grains, potatoes, fruit juices, sugar-sweetened beverages, and sweets/dessert), and animal foods (Animal fats, eggs, dairy, meat (poultry and red meat), fish/seafood, and miscellaneous animal-based foods). All food groups were categorized into quintiles. Positive or reverse scores (1–5) were assigned to each quintile of food groups, and indices were created by adding up the scores. To calculate PDI, all plant food groups and animal food groups were assigned positive and reverse scores, respectively. Regarding hPDI, healthy plant food groups received positive scores, and less healthy plant food groups and animal food groups received inverse scores. Finally, we calculate uPDI by assigning positive scores for less healthy plant food groups and inverse scores for healthy plant food groups and animal food groups. Food group scores were added up to create the indices. Then, we divided each index into tertiles.

### Anthropometry assessment

To assess the anthropometric measurements of the study population, weight, height, and mid-upper arm circumference (MUAC) were measured for each child. Weight was measured using a digital scale with a precision of 100 g. The height was measured using plastic tape attached to the wall with a precision of 0.1 cm. MUAC was measured with tape at the point between the shoulder and elbow. The formula for calculating BMI is weight in kilograms divided by height in meters squared. BMI-for-age (BAZ), height-for-age (HAZ), and weight-for-age (WAZ) z-scores were calculated using the guidelines of the World Health Organization [25]. HAZ was divided into short stature (HAZ ≤ −2 SD) and severe short stature (HAZ ≤ −3 SD). Categories for WAZ: -3 SD < WAZ <-1 SD considered underweight, and WAZ ≤ −2 SD considered severely underweighted. BAZ categories are obese (BAZ ≥ 2 SD), overweight (BAZ ≥ 1 SD), normal (BAZ ≥ −1 SD and < −1 SD), and underweight (BAZ ≤ −2 SD).

### Assessment of covariates

Socioeconomic Status (SES) includes education, the job of parents, house ownership or tenancy, number of family members, car ownership, number of cars, number of bedrooms in the house, and having appliances such as washing machine, dishwasher, vacuum cleaner, LCD TV, side-by-side refrigerator, computer, laptop, air conditioner, and advanced heating system was assessed using a valid and reliable questionnaire. We gave a score to each questionnaire item and then summed it to calculate the total socioeconomic status score. Finally, the scores were separated into three groups for qualitative description: weak, moderate, and rich. Physical activity was assessed using the International Physical Activity Questionnaire (IPAQ) short form [26].

### Validity and reliability of food frequency questionnaire

To assess the reliability of the questionnaire used in this study, 56 parents completed it twice, 12 weeks apart, and the intra-class correlation coefficient to determine reproducibility was applied. The validity was assessed by collecting three 24-hour recalls during the study and using Pearson correlations and Wilcoxon signed-rank test. The strength of the correlation for both reliability and validity data interpreted through the following rating interpretations were used: for Pearson statistics, 0.10 to 0.30 was considered weak, 0.30 to 0.50 moderate, and >0.50 strong [27]; for ICC statistics, 0.00 to 0.10 was considered none, 0.11 to 0.40 slight, 0.41 to 0.60 fair, 0.61 to 0.80 moderate, and 0.81 to 0.1 substantial [28].

### Statistical analysis

We divided all participants into tertile ranges based on PDI, hPDI, and uPDI scores. To evaluate the association between tertiles of PDI, hPDI, and uPDI and anthropometric parameters, a one-way analysis of variance (ANOVA) was used. We used an analysis of covariance (ANCOVA) to assess the association between dietary intakes and anthropometric measures across PDI, hPDI, and uPDI tertiles. The Chi-square test was applied to assess the distribution of individuals in terms of descriptive characteristics (i.e., supplement use, socioeconomic level, etc.) throughout the groups. We used binary logistic regression to investigate the association between PDI, hPDI, and uPDI with underweight, overweight, and obesity. Three models were applied in the analysis: Model 1: an unadjusted model; Model 2: adjusted for age and energy; and Model 3: adjusted for age, energy, parent education, parent occupation, supplement use, parent smoking, physical activity, and socioeconomic status. All the statistical analyses were performed using SPSS (SPSS Inc., version 19). A p-value <0.05 was considered statistically significant.

## Results

Demographic characteristics of 330 primary school girls across tertiles of PDI, hPDI, and uPDI scores are presented in Tables 1 and 2. There was a significant association between PDI and uPDI with anthropometric indices (e.g., weight, height, BMI, and MUAC). Additionally, a significant association was observed between PDI score and age. Mothers of participants in the highest tertile of uPDI were less likely to be university graduates. Conversely, there was a positive association between PDI score and the education level of the child’s father. Most of the participants have low levels of physical activity.

**Table 1:** Demographic characteristics of primary school girls across tertiles of PDI, hPDI, and uPDI scores (Quantitative variables).

	Tertiles of PDI				Tertiles of hPDI				Tertiles of uPDI			
Variable	Tertile 1 ≤50 N = 111	Tertile 2 >50 <57 N = 101	Tertile 3 57 ≤ N = 118	P value	Tertile 1 ≤51 N = 111	Tertile 2 >51 <57 N = 93	Tertile 3 57≤ N = 126	P value	Tertile 1 ≤49 N = 123	Tertile 2 >49 <57 N = 90	Tertile 3 57≤ N = 117	P value
Age (year) Weight (kg) Height (cm) BMI (kg/m2) MUAC (cm) Physical activity (met/min/week)	8.83(1.79) 30.75(12.69) 134.08(13.63) 16.47(3.76) 20.99(3.52) 163.34(346.08)	8.81(1.75) 33.47(12.95) 136.91(13.71) 17.24 (3.72) 22.00(3.51) 180.99(486.24)	9.38(1.83) 36.97(12.81) 138.93(13.05) 18.66 (3.98) 22.88(3.87) 233.61(445.57)	0.026 0.001 0.025 <0.001 0.001 0.435	8.90(1.83) 35.06(14.75) 137.18(13.69) 17.88(4.37) 22.24(4.14) 222.31(555.93)	9.09(1.77) 34.75(12.35) 137.74(12.78) 17.81(3.90) 22.36(3.48) 189.18(370.41)	9.08(1.82) 32.01(11.75) 135.46(14.02) 16.90(3.46) 21.45(3.45) 172.27(329.63)	0.693 0.142 0.420 0.103 0.130 0.665	9.22(1.75) 37.20(14.41) 139.23(12.97) 18.55(4.35) 23.09(3.94) 239.31(452.30)	9.09(1.91) 35.04(12.17) 138.35(13.11) 17.80(3.75) 22.06(3.60) 206.33(497.56)	8.76(1.77) 29.29(10.76) 132.70(13.71) 16.13(3.15) 20.72(3.17) 136.52(330.98)	0.134 <0.001 <0.001 <0.001 <0.001 0.169

*The P value reported for the quantitative variables was resulted from one-way ANOVA, and the numbers are reported as mean ± SD. *P value <0.05 shows a significant level of association. BMI body mass index, MET metabolic equivalents, PDI overall plant-based diet index, hPDI healthful plant-based diet index, uPDI unhealthful plant-based diet index.

**Table 2:** Demographic characteristics of primary school girls across tertiles of PDI, hPDI, and uPDI scores (Qualitative variables).

		Tertiles of PDI				Tertiles of hPDI				Tertiles of uPDI			
Variable		Tertile 1 ≤50 N = 111	Tertile 2 >50 <57 N = 101	Tertile 3 57 ≤ N = 118	P value	Tertile 1 ≤51 N = 111	Tertile 2 >51 <57 N = 93	Tertile 3 57≤ N = 126	P value	Tertile 1 ≤49 N = 123	Tertile 2 >49 <57 N = 90	Tertile 3 57≤ N = 117	P value
Grade	1st 2nd 3rd 4th 5th 6th	22 (6.7%) 21 (6.4%) 20 (6.1%) 17 (5.2%) 12 (3.6%) 19 (5.8%)	20 (6.1%) 15 (4.5%) 15 (4.5%) 18 (5.5%) 22 (6.7%) 11 (3.3%)	16 (4.8%) 15 (4.5%) 20 (6.1%) 18 (5.5%) 23 (7.0%) 26 (7.9%)	0.289	23 (7.0%) 16 (4.8%) 14 (4.2%) 23 (7.0%) 18 (5.5%) 17 (5.2%)	11 (3.3%) 17 (5.2%) 20 (6.1%) 13 (3.9%) 15 (4.5%) 17 (5.2%)	24 (7.3%) 18 (5.5%) 21 (6.4%) 17 (5.2%) 24 (7.3%) 22 (6.7%)	0.577	15(4.5%) 17(5.2%) 18(5.5%) 27(8.2%) 25(7.6%) 21(6.4%)	17(5.2%) 13(3.9%) 14(4.2%) 14(4.2%) 12(3.6%) 20(6.1%)	26(7.9%) 21(6.4%) 23(7.0%) 12(3.6%) 20(6.1%) 15(4.5%)	0.150
Socioeconomic status	Low Medium High	73 (22.1%) 38 (11.5%) 0 (0.0%)	63 (19.1%) 37 (11.2%) 1 (0.3%)	50 (15.2%) 62 (18.8%) 6 (1.8%)	0.001	57 (17.3%) 52 (15.8%) 2 (0.6%)	56 (17.0%) 36 (10.9%) 1 (0.3%)	73 (22.1%) 49(14.8%) 4 (1.2%)	0.536	64(19.4%) 56(17.0%) 3 (0.9%)	43(13.0%) 43(13.0%) 4 (1.2%)	79(23.9%) 38(11.5%) 0 (0.0%)	0.013
Supplement	Yes No	11 (3.3%) 100 (30.3%)	19 (5.8%) 82 (24.8%)	30 (9.1%) 88 (26.7%)	0.010	21 (6.4%) 90 (27.3%)	20 (6.1%) 73 (22.1%)	19 (5.8%) 107 (32.4%)	0.462	26(7.9%) 97(29.4%)	19(5.8%) 71(21.5%)	15(4.5%) 102(30.9%)	0.174
Parents smoking	Yes No	12 (3.6%) 99 (30.0%)	8 (2.4%) 93 (28.2%)	18 (5.5%) 100 (30.3%)	0.228	11 (3.3%) 100 (30.3%)	11 (3.3%) 82 (24.8%)	16 (4.8%) 110 (33.3%)	0.793	14(4.2%) 109(33.0%)	11(3.3%) 79(23.9%)	13(3.9%) 104(31.5%)	0.968
Physical activity	Low Medium High	103 (31.2%) 8 (2.4%) 0 (0.0%)	94 (28.5%) 6 (1.8%) 1 (0.3%)	104 (31.5%) 14 (4.2%) 0 (0.0%)	0.284	98 (29.7%) 12 (3.6%) 1 (0.3%)	85 (25.8%) 8 (2.4%) 0 (0.0%)	118 (36.4%) 8 (2.4%) 0 (0.0%)	0.471	105(31.8%) 18(5.5%) 0(0.0%)	83(25.2%) 6(1.8%) 1(0.3%)	113(34.2%) 4(1.2%) 0(0.0%)	0.012
Father’s education	High school Diploma College	30 (9.1%) 51 (15.5%) 30 (12.1%)	13 (3.9%) 53 (16.1%) 35 (10.6%)	16 (4.8%) 58 (17.6%) 44 (13.3%)	0.037	14 (4.2%) 59 (17.9%) 38 (11.5%)	18 (5.5%) 40 (12.1%) 35 (10.6%)	27 (8.2%) 63 (19.1%) 36 (10.9%)	0.265	21(6.4%) 58(17.6%) 44(13.3%)	12(3.6%) 43(13.0%) 35(10.6%)	26(7.9%) 61(18.5%) 30(9.1%)	0.213
Mother’s education	High school Diploma College	21 (6.4%) 59 (17.9%) 31 (9.4%)	11 (3.3%) 52 (15.8%) 38 (11.5%)	11 (3.3%) 65 (19.7%) 42 (12.7%)	0.173	11 (3.3%) 56 (17.0%) 44 (13.3%)	13 (3.9%) 47 (14.2%) 33 (10.0%)	19 (5.8%) 73 (22.1%) 34 (10.3%)	0.282	10(3.0%) 62(18.8%) 51(15.5%)	10(3.0%) 46(13.9%) 34(10.3%)	23(7.0%) 68(20.6%) 26(7.9%)	0.006
Family member	4 or less 5 to 7 More than 7	72 (21.8%) 37 (11.2%) 2 (0.6%)	75 (22.7%) 23 (7.0%) 3 (0.9%)	81 (24.5%) 36 (10.9%) 1 (0.3%)	0.382	78 (23.6%) 30 (9.1%) 3 (0.9%)	67 (20.3%) 23 (7.0%) 3 (0.9%)	83 (25.2%) 43 (13.0%) 0 (0.0%)	0.201	87(26.4%) 33(10.0%) 3(0.9%)	64(19.4%) 24(7.3%) 2(0.6%)	77(23.3%) 39(11.8%) 1(0.3%)	0.673
Father’s occupation	Manager Employee Worker Housewife Retired Self-employment Others	2 (0.6%) 25 (7.6%) 14 (4.2%) 0 (0.0%) 1 (0.3%) 67 (20.3%) 2 (0.6%)	3 (0.9%) 29 (8.8%) 8 (2.4%) 1 (0.3%) 0 (0.0%) 58 (17.6%) 2 (0.6%)	3 (0.9%) 39 (11.8%) 8 (2.4%) 0 (0.0%) 2 (0.6%) 64 (19.4%) 2 (0.6%)	0.675	2 (0.6%) 34 (10.3%) 7 (2.1%) 0 (0.0%) 0 (0.0%) 66 (20.0%) 2 (0.6%)	3 (0.9%) 25 (7.6%) 7 (2.1%) 0 (0.0%) 0 (0.0%) 57 (17.3%) 1 (0.3%)	3 (0.9%) 34 (10.3%) 16 (4.8%) 1 (0.3%) 3 (0.9%) 66 (20.0%) 3 (0.9%)	0.475	3(0.9%) 38(11.5%) 10(3.0%) 1(0.3%) 1(0.3%) 68(20.6%) 2(0.6%)	2(0.6%) 37(11.2%) 6(1.8%) 0(0.0%) 2(0.6%) 42(12.7%) 1(0.3%)	3(0.9%) 18(5.5%) 14(4.2%) 0(0.0%) 0(0.0%) 79(23.9%) 3(0.9%)	0.025
Mother’s occupation	Manager Employee Worker Housewife Retired Self-employment Others	0 (0.0%) 11 (3.3%) 1 (0.3%) 93 (28.2%) 0 (0.0%) 5 (1.5%) 1 (0.3%)	1 (0.3%) 9 (2.7%) 0 (0.0%) 76 (23.0%) 1 (0.3%) 11 (3.3%) 3 (0.9%)	1 (0.3%) 11 (3.3%) 0 (0.0%) 96 (29.1%) 0 (0.0%) 8 (2.4%) 2 (0.6%)	0.605	0 (0.0%) 11 (3.3%) 1 (0.3%) 86 (26.1%) 1 (0.3%) 10 (3.0%) 2 (0.6%)	1 (0.3%) 7 (2.1%) 0 (0.0%) 75 (22.7%) 0 (0.0%) 6 (1.8%) 4 (1.2%)	1 (0.3%) 13 (3.9%) 0 (0.0%) 104 (31.5%) 0 (0.0%) 8 (2.4%) 0 (0.0%)	0.458	0(0.0%) 9(2.7%) 0(0.0%) 100(30.3%) 1(0.3%) 10(3.0%) 3(0.9%)	1(0.3%) 11(3.3%) 0(0.0%) 70(21.2%) 0(0.0%) 6(1.8%) 2(0.6%)	1(0.3%) 11(3.3%) 1(0.3%) 95(28.8%) 0(0.0%) 8(2.4%) 1(0.3%)	0.836

*The P value for the qualitative variables was calculated by the chi-square test, and the results are based on N (%). *P value <0.05 shows a significant level of association. PDI overall plant-based diet index, hPDI healthful plant-based diet index, uPDI unhealthful plant-based diet index.

Table 3 represents participants’ dietary intakes across tertiles of PDI, hPDI, and uPDI scores. Higher PDI was significantly associated with higher intakes of energy, fiber, PUFA, vitamin K, E, B6, and B9, as well as food groups including vegetables, beans, fats, chocolate, and snacks. It was also associated with lower intake of cholesterol, vitamin B2, B12, D, calcium, zinc, and meat and dairy food group. Participants in the highest tertile of hPDI had higher intakes of fiber, vitamins E, K, B1, selenium, beans, and chocolate and snacks. They also consumed lower energy, cholesterol, MUFA, vitamin D, B2, B12, zinc, meat, dairy, and added sugar beverages. Moreover, the highest uPDI was accompanied by lower intakes of energy, fiber, cholesterol, SFA, MUFA, vitamins A, D, K, B2, B3, B5, B9, B12, calcium, magnesium, potassium, zinc, iron, phosphorus, vegetables, meat, nuts and seeds, dairy, and fats.

**Table 3:** Dietary intakes of primary school girls across tertiles of PDI, hPDI, and uPDI scores.

	Tertiles of PDI				Tertiles of hPDI				Tertiles of uPDI				
Variable	Tertile 1 ≤50 N = 111	Tertile 2 >50 <57 N = 101	Tertile 3 57 ≤ N = 118	P value	Tertile 1 ≤51 N = 111	Tertile 2 >51 <57 N = 93	Tertile 3 57≤ N = 126	P value	Tertile 1 ≤49 N = 123	Tertile 2 >49 <57 N = 90	Tertile 3 57≤ N = 117	P value	
Energy (kcal/d) Fiber (g/d) Cholesterol (mg/d) SFA (g/d) MUFA (g/d) PUFA (g/d)	1367.51(470.77) 13.43(0.26) 275.40(6.86) 22.40(0.68) 19.56(0.34) 13.53(0.32)	1865.67(481.09) 13.72(0.24) 268.44(6.33) 21.98(0.63) 19.78(0.31) 13.48(0.29)	2276.21(574.74) 15.19(0.24) 242.84(6.55) 20.36(0.65) 18.89(0.32) 14.78(0.30)	<0.001 <0.001 0.004 0.103 0.140 0.006	2160.15(576.02) 13.38(0.24) 294.54(5.92) 21.69(0.63) 20.25(0.31) 13.72(0.30)	1899.86(496.05) 14.04(0.25) 266.91(6.17) 22.18(0.66) 19.64(0.32) 13.87(0.31)	1526.62(634.04) 14.90(0.22) 228.73(5.59) 20.94(0.59) 18.45(0.29) 14.25(0.28)	<0.001 <0.001 <0.001 0.383 <0.001 0.455	2335.57(521.15) 14.80(0.26) 310.99(6.02) 23.03(0.67) 20.61(0.33) 14.55(0.32)	1848.89(471.86) 14.15(0.26) 261.20(5.93) 20.60(0.66) 19.09(0.33) 13.52(0.31)	1326.02(408.95) 13.46(0.27) 210.07(6.23) 20.70(0.69) 18.32(0.34) 13.69(0.33)		<0.001 0.009 <0.001 0.029 <0.001 0.068
Vitamin A Vitamin D (μg) Vitamin E (mg) Vitamin K (mg) Thiamine (mg) Riboflavin (mg) Niacin (mg) Vitamin B5 (mg) Vitamin B6 (mg) Folic acid (μg) Vitamin B12 (μg) Vitamin C (mg)	779.19(49.51) 1.48(0.11) 11.08(0.30) 158.46(4.93) 1.61(0.02) 1.53(0.03) 16.41(0.20) 5.14(0.08) 1.41(0.04) 206.85(4.57) 3.60(0.08) 96.38(4.55)	739.17(45.64) 1.60(0.10) 11.19(0.27) 160.91(4.55) 1.61(0.02) 1.54(0.02) 16.20(0.19) 5.09(0.07) 1.45(0.03) 208.12(4.21) 3.50(0.07) 94.96(4.19)	719.03(47.29) 1.21(0.10) 12.78(0.28) 177.76(4.71) 1.63(0.02) 1.39(0.02) 15.96(0.19) 4.92(0.07) 1.59(0.03) 224.07(4.36) 2.89(0.08) 100.66(4.34)	0.720 0.027 <0.001 0.014 0.864 <0.001 0.360 0.158 0.009 0.015 <0.001 0.633	714.11(45.64) 1.62(0.10) 10.86(0.27) 157.61(4.35) 1.53(0.02) 1.53(0.02) 16.32(0.19) 4.98(0.07) 1.49(0.03) 207.26(4.24) 3.66(0.07) 95.27(4.19)	780.15(47.59) 1.48(0.10) 11.62(0.29) 169.36(5.20) 1.60(0.02) 1.55(0.02) 16.20(0.19) 5.09(0.07) 1.47(0.04) 213.14(4.42) 3.49(0.07) 94.62(4.37)	747.40(43.15) 1.21(0.09) 12.55(0.26) 171.93(4.16) 1.71(0.02) 1.39(0.02) 16.07(0.18) 5.07(0.07) 1.51(0.03) 218.99(4.01) 2.87(0.07) 101.53(3.96)	0.601 0.017 <0.001 0.056 <0.001 <0.001 0.665 0.555 0.758 0.161 <0.001 0.444	823.36(48.45) 1.77(0.10) 12.16(0.30) 179.49(4.77) 1.60(0.02) 1.63(0.02) 16.51(0.20) 5.30(0.07) 1.52(0.04) 224.29(4.53) 3.72(0.08) 101.59(4.50)	808.38(47.72) 1.32(0.10) 11.35(0.30) 170.35(4.87) 1.62(0.02) 1.47(0.02) 16.34(0.20) 4.92(0.07) 1.54(0.04) 210.49(4.46) 3.27(0.08) 99.18(4.43)	615.08(50.14) 1.13(0.11) 11.54(0.31) 150.69(4.59) 1.64(0.02) 1.35(0.03) 15.73(0.21) 4.87(0.08) 1.42(0.04) 204.18(4.69) 2.92(0.08) 91.83(4.66)		0.010 0.001 0.167 <0.001 0.643 <0.001 0.048 0.001 0.110 0.020 <0.001 0.379
Calcium (mg) Magnesium (mg) Potassium (mg) Zinc (mg) Fe (mg) Phosphorus (mg) Selenium (mg)	780.63(22.72) 216.16(3.07) 2755.84(45.20) 8.26(0.12) 13.68(0.20) 1088.62(18.20) 0.10(0.002)	779.13(20.94) 220.01(2.83) 2815.61(41.67) 8.33(0.11) 13.51(0.18) 1091.99(16.78) 0.10(0.002)	692.27(21.70) 219.46(2.93) 2848.75(43.17) 7.79(0.12) 13.75(0.19) 1039.85(17.38) 0.09(0.002)	0.008 0.644 0.394 0.004 0.646 0.077 0.683	737.01(21.07) 215.00(2.81) 2797.28(41.69) 8.38(0.11) 13.73(0.18) 1077.66(16.81) 0.09(0.002)	793.71(21.97) 223.15(2.93) 2857.92(43.46) 8.38(0.11) 13.64(0.19) 1097.87(17.52) 0.10(0.002)	725.45 (19.92) 218.20(2.66) 2778.92(39.41) 7.68(0.10) 13.59(0.17) 1048.48(15.89) 0.10(0.002)	0.052 0.127 0.377 <0.001 0.872 0.117 0.037	797.09(22.64) 230.55(2.94) 2983.70(43.38) 8.66(0.12) 14.04(0.20) 1129.35 (17.81) 0.10(0.002)	731.46(22.30) 214.30(2.90) 2784.80(42.72) 7.99(0.11) 13.87(0.19) 1052.47(17.54) 0.09(0.002)	710.73(23.43) 209.13(3.05) 2639.31(44.89) 7.63(0.12) 13.08(0.20) 1027.33(18.43) 0.10(0.002)		0.045 <0.001 <0.001 <0.001 0.007 0.001 0.406
*Grains (g)* *Fruits (g)* *Vegetables (g)* *Meat and its products (g)* *Beans (g)* *Nuts and seeds (g)* *Dairy products (g)* *Fats (g)* *Added sugar beverages(g)* *Chocolate and snacks (g)*	119.18(4.78) 265.39(13.04) 264.10(7.47) 86.15(2.70) 33.02(1.54) 14.58(1.25) 419.84(18.81) 12.19(0.40) 76.06(8.81) 150.68(13.74)	115.32(4.35) 275.25(11.93) 273.18(6.89) 82.40(2.49) 35.38(1.42) 13.56(1.15) 427.80(17.34) 11.71(0.36) 86.88(8.12) 175.69(12.66)	123.86(4.54) 298.89(12.45) 292.48(7.14) 67.71(2.58) 40.97(1.47) 16.26(1.20) 341.57(17.96) 13.02(0.38) 98.85(8.42) 252.93(13.19)	0.393 0.211 0.036 <0.001 0.002 0.266 0.002 0.049 0.237 <0.001	116.30(4.37) 276.28(12.02) 265.08(6.91) 95.42(2.25) 33.53(1.44) 14.02(1.15) 408.88(17.49) 12.43(0.37) 103.25(8.05) 149.52(12.84)	124.99(4.57) 271.66(12.55) 285.43(7.21) 79.74(2.34) 37.44(1.50) 13.52(1.20) 424.86(18.23) 12.27(0.38) 93.08(8.40) 207.49(13.47)	118.69(4.13) 290.32(11.32) 281.34(6.53) 62.43(2.12) 38.65(1.36) 16.61(1.09) 358.87(16.53) 12.31(0.35) 69.55(7.61) 225.24(12.14)	0.357 0.525 0.099 <0.001 0.038 0.134 0.023 0.952 0.012 <0.001	113.82(4.67) 298.86(12.90) 303.14(7.26) 90.53(2.63) 40.11(1.54) 15.02(1.25) 438.66(18.80) 13.98(0.38) 77.51(8.75) 195.03(14.10)	125.85(4.65) 274.69(12.64) 277.28(7.15) 79.97(2.59) 35.67(1.52) 15.00(1.23) 383.91(18.51) 11.63(0.38) 87.56(8.61) 175.54(13.83)	121.15(4.86) 265.36(13.35) 249.38(7.51) 64.46(2.72) 33.59(1.60) 14.61(1.29) 355.63(19.45) 11.15(0.39) 98.02(9.05) 209.57(14.77)		0.190 0.249 <0.001 <0.001 0.029 0.973 0.022 <0.001 0.361 0.220

SFA = saturated fatty acids, MUFA = monounsaturated fatty acids, and PUFA = polyunsaturated fatty acids. *The P value is reported from covariance analysis, and the results are based on mean ± SD. *All of the variables are adjusted for energy intake. *P value <0.05 shows a significant level of association. PDI overall plant-based diet index, hPDI healthful plant-based diet index, uPDI unhealthful plant-based diet index.

The mean values and standard deviation or standard error that were calculated for the distribution of anthropometric measures across tertiles of PDI, hPDI, and uPDI scores in the three statistical models are displayed in Table 4. There was a significant positive association between PDI score and anthropometric indices (MUAC (22.88±3.87 vs. 20.99±3.52 kg; P = 0.001), BAZ (0.63±1.39 vs. -0.27±1.51 kg; P = <0.001) and HAZ (0.60±1.15 vs. 0.35±1.33 kg; P = 0.018)) in the crude model. In model 2, with an increase in PDI score, a significant decrease was seen in HAZ (0.38±0.12 vs. 0.59±0.12 kg; P = 0.033).

**Table 4:** Distribution of anthropometric measures across tertiles of PDI, hPDI, and uPDI scores.

Tertiles of PDI						Tertiles of hPDI				Tertiles of uPDI			
Variable		Tertile 1 ≤50 N = 111	Tertile 2 >50 <57 N = 101	Tertile 3 57 ≤ N = 118	P value	Tertile 1 ≤51 N = 111	Tertile 2 >51 <57 N = 93	Tertile 3 57≤ N = 126	P value	Tertile 1 ≤49 N = 123	Tertile 2 >49 <57 N = 90	Tertile 3 57≤ N = 117	P value
MUAC (cm)	Model 1 Model 2 Model 3	20.99(3.52) 21.93(0.32) 21.95(0.32)	22.00(3.51) 22.19(0.29) 22.12(0.29)	22.88(3.87) 21.82(0.30) 21.86(0.30)	0.001 0.653 0.30	22.24(4.14) 21.90(0.29) 21.87(0.29)	22.36(3.48) 22.21(0.30) 22.18(0.30)	21.45(3.45) 21.85(0.27) 21.90(0.28)	0.130 0.655 0.714	23.09(3.94) 22.29(0.31) 22.17(0.32)	22.06(3.60) 21.98(0.31) 22.02(0.31)	20.72(3.17) 21.63(0.32) 21.73(0.33)	<0.001 0.442 0.703
BAZ	Model 1 Model 2 Model 3	-0.27(1.51) -0.02(0.14) 0.06(0.14)	0.16(1.30) 0.18(0.13) 0.15(0.13)	0.63(1.39) 0.33(0.14) 0.32(0.14)	<0.001 0.398 0.497	0.34(1.43) 0.14(0.13) 0.13(0.13)	0.32(1.44) 0.27(0.14) 0.26(0.14)	-0.06(1.46) 0.15(0.12) 0.17(0.12)	0.053 0.745 0.802	0.56(1.42) 0.25(0.14) 0.21(0.14)	0.35(1.37) 0.33(0.14) 0.33(0.14)	-0.34(1.395) -0.006(0.15) 0.04(0.15)	<0.001 0.251 0.378
HAZ	Model 1 Model 2 Model 3	0.35(1.33) 0.59(0.12) 0.58(0.12)	0.83(1.15) 0.82(0.11) 0.79(0.11)	0.60(1.15) 0.38 (0.12) 0.42(0.12)	0.018 0.033 0.077	0.79(1.18) 0.68(0.12) 0.66(0.11)	0.70(1.07) 0.68(0.12) 0.68(0.12)	0.32(1.32) 0.44(0.11) 0.45(0.11)	0.008 0.266 0.321	0.80(1.08) 0.68(0.12) 0.61(0.12)	0.81(1.27) 0.81(0.12) 0.83(0.12)	0.18(1.24) 0.32(0.13) 0.37(0.13)	<0.001 0.028 0.035
WAZ	Model 1 Model 2 Model 3	-0.09(1.38) 0.36(1.00) 0.50(1.03)	0.43(1.19) 0.43(0.89) 0.34(0.91)	-0.79(14.31) -1.29(1.05) -1.33(1.07)	0.635 0.433 0.439	0.55(1.39) 0.57(0.93) 0.60(0.95)	0.58(1.29) 0.60(0.98) 0.81(1.00)	-1.29(13.03) -1.32(0.89) -1.50(0.92)	0.222 0.269 0.195	0.68(1.34) 0.76(1.02) 0.90(1.06)	-1.23(16.03) -1.31(1.03) -1.65(1.06)	-0.19(1.20) 0.21(0.99) -0.12(1.02)	0.360 0.352 0.220

*The model 1 was resulted from one-way ANOVA, and the numbers are reported as mean ± SD. *Model 2 was resulted from covariance analysis, and the numbers are reported as mean ± SE. *P value <0.05 shows a significant level of association. Model 1: crude. Model 2: Adjusted for age and energy. Model 3: Adjusted for age, energy, parents’ education, parents’ occupation, supplement use, parents’ smoking, physical activity and socio-economic status. PDI overall plant-based diet index, hPDI healthful plant-based diet index, uPDI unhealthful plant-based diet index.

In the crude model, no association was observed between **h**PDI scores with MUAC, BAZ, and WAZ (P≥0.05); however, a significant inverse association was observed between HAZ (0.32±1.32 vs. 0.79±1.18 kg; P = 0.008) and **h**PDI scores. After adjustment for confounders, no significant association was found between tertiles of **h**PDI with anthropometric indices.

The participants in the highest tertiles of uPDI had significantly lower anthropometric measures, including MUAC (uPDI: 20.72±3.17 vs. 23.09±3.94 cm; P = <0.001), BAZ (uPDI: -0.34±1.39 vs. 0.56±1.42; P = <0.001), and HAZ (uPDI: 0.18±1.24 vs. 0.80±1.08; P = <0.001) in the crude model. A significant decrease was observed in HAZ in models 2 (P = 0.028) and 3 (P = 0.035). The association between uPDI and MUAC, and BAZ disappeared after adjustment. No significant association was found between the tertiles of uPDI and WAZ (P≥0.05).

Crude and multivariable-adjusted ORs and 95% CIs for being underweight, overweight, and obese across tertiles of PDI, hPDI, and uPDI scores are presented in Table 5. In model 1 and model 2, children in the highest tertile of PDI had lower odds of being underweight (Model 1: OR: 4.11; 95% CI: 1.31–12.90; P trend = 0.007; model 2: OR: 3.89; 95% CI: 1.22–12.37; P trend = 0.010) and lower odds of being overweight (Model 1: OR: 0.35; 95% CI: 0.19–0.62; P trend = <0.001; model 2: OR: 0.37; 95% CI: 0.20–0.68; P trend = 0.001). In model 3, the association between PDI score and overweight remained significant (P trend = 0.001), but the association between PDI score and underweight reversed (OR: 4.00; 95% CI: 0.55–28.73; P trend = 0.04). Higher hPDI scores were associated with decreased underweight risk in the crude model and adjusted models. (Model 1: P trend = 0.021; model 2: P trend = 0.018; model 3: P trend = 0.031). No significant association was found between tertiles of hPDI and odds of being overweight (P trend≥0.05). There was a significant positive association between uPDI and odds of being overweight and obese in all three models (Model 1: P trend<0.001; Model 2: P trend<0.001; Model 3: P trend = 0.001). No significant association was observed between underweight and tertiles of uPDI scores after adjustment (P trend≥0.05).

**Table 5:** Crude and multivariable-adjusted ORs and 95% CIs for being underweight, overweight and obesity across tertiles of PDI, hPDI, and uPDI scores.

		Tertiles of PDI				Tertiles of hPDI						Tertiles of uPDI				
Variable		T1 ≤50 N = 111	T2 >50 <57 N = 101	T3 57 ≤ N = 118	P value	T1 ≤51 N = 111	T2 >51 <57 N = 93	T3 57≤ N = 126			P value	T1 ≤49 N = 123	T2 >49 <57 N = 90	T3 57≤ N = 117		P value
BAZ																
Underweight	Model 1 Model 2 Model 3	1 1 1	4.71(1.31–16.92) 4.72(1.28–17.30) 3.70(0.68–20.00)	4.11(1.31–12.90) 3.89(1.22–12.37) 4.00(0.55–28.73)	0.007 0.010 0.040	1 1 1	0.48(0.11–2.10) 0.48(0.11–2.12) 1.16(0.15–8.62)		0.24(0.06–0.87) 0.24(0.06–0.89) 0.45(0.07–2.81)	0.021 0.018 0.031		1 1 1	1.48(0.36–6.11) 1.56(0.37–6.51) 1.58(0.19–12.82)		0.44(0.16–1.23) 0.49(0.17–1.38) 0.97(0.21–4.37)	0.097 0.122 0.290
Overweight and obesity	Model 1 Model 2 Model 3	1 1 1	0.62(0.33–1.15) 0.59(0.31–1.13) 0.64(0.28–1.43)	0.35(0.19–0.62) 0.37(0.20–0.68) 0.36(0.16–0.79)	<0.001 0.001 0.001	1 1 1	1.07(0.60–1.91) 1.10(0.60–1.99) 1.21(0.56–2.61)		1.46(0.84–2.53) 1.51(0.85–2.68) 1.86(0.86–4.01)	0.171 0.123 0.144		1 1 1	1.64(0.93–2.90) 1.71(0.95–3.09) 1.49(0.65–3.42)		3.03(1.71–5.37) 2.90(1.61–5.23) 2.85(1.33–6.09)	<0.001 <0.001 0.001

Odds ratio and 95% confidence interval for underweight in tertiles of PDI, hPDI, and uPDI scores. Underweight defined as BAZ< −2 SD and overweight and obesity defined as BAZ ≥ 1 SD. Model 1: crude. Model 2: Adjusted for age and calorie. Model 2: Adjusted for age, calorie, parents’ education, parents’ occupation, supplement use, parents’ smoking, physical activity and socio-economic status. PDI overall plant-based diet index, hPDI healthful plant-based diet index, uPDI unhealthful plant-based diet index.

The results of the reliability and reproducibility of the food frequency questionnaire are presented in Table 6. The findings indicate that the newly designed FFQ is valid and reliable. The correlation coefficients between the FFQ and 24-hour dietary recalls for carbohydrates, proteins, and fat were 0.52, 0.54, and 0.51, respectively. Furthermore, most nutrient values did not show significant differences between the FFQ and 3-day dietary records, according to the Wilcoxon signed-rank test (P>0.05). The intra-class correlation coefficients ranged from 0.54 to 0.77, indicating that the FFQ is consistent. Overall, this FFQ can be considered a valuable tool for evaluating dietary intake in this population.

**Table 6:** Reproducibility and validation study: Correlation coefficient and Wilcoxon signed-rank test for validity and ICC for reproducibility.

	FFQ	3DR	Wilcoxon Signed Rank Test (p Value)^1^	Correlation coefficient^2^	ICC^3^
	Mean ± SD	Mean ± SD			
Carbohydrate	262.22(92.28)	235.75(84.97)	0.013	0.7	0.52
Protein	63.42(22.62)	58.75(20.94)	0.059	0.63	0.54
Fat	64.02(25.76)	60.28(24.58)	0.136	0.61	0.51
Calcium	748.58(335.10)	696.48(272.37)	0.434	0.54	0.59
Fe	13.65(5.01)	12.77(4.78)	0.016	0.75	0.74
Magnesium	218.52(76.82)	201.07(81.82)	0.013	0.65	0.60
Vitamin C	97.48(54.55)	95.75(62.34)	0.211	0.77	0.62
Vitamin A	777.25(503.65)	745.43(532.87)	0.915	0.68	0.70
Vegetables	170.65(87.34)	175.79(107.75)	0.866	0.59	0.75
Fruits	280.38(155.49)	284.13(170.27)	0.135	0.68	0.65
Meat and its products	89.56(41.06)	78.41(34.25)	0.124	0.68	0.75
Dairy products	394.29(224.29)	370.29(192.16)	0.541	0.89	0.62

^1^ Wilcoxon signed-rank test were used to examine the difference between FFQ and 3DR with a significant p value<0.001.

^2^Pearson or Spearman correlation coefficient were used to assess the correlation between variables with a p-value < 0.05 considered as significant.

^3^the intra-class coefficient for the comparison between FFQ1 and FFQ2. 3DR, dietary recall.

## Discussion

To the best of our knowledge, this study is the first investigation to examine the association between adherence to plan-based dietary patterns and anthropometry indices in Kerman. In this cross-sectional study, we found that higher uPDI scores were significantly associated with decreased HAZ and increased risk of overweight and obesity, while higher PDI scores were associated with a lower risk of being overweight and higher hPDI scores were associated with decreased risk of being underweight.

There have been few studies conducted on children to evaluate the effect of plant-based dietary patterns on their growth. Therefore, we can compare our findings with studies that assess the association between vegetarian/ vegan diets or animal and plant-based components with anthropometry changes.

This study found an inverse association between uPDI scores and HAZ. In a cross-sectional study on children aged 5–10, vegetarians and vegans had lower HAZ than omnivores [29]. In another study, vegetarian adolescent boys (12–17-y-old) were shorter, while no significant difference was found in vegetarian children (6–9-y-old girls and 6–11-y-old boys) [30]. Studies have shown a positive association between animal-based protein and height [31,32]. Although uPDI is defined as a higher amount of animal-based protein, it contains high consumption of refined grains, sweets, snack foods, added fats, and non-water beverages, particularly in children, which can result in lower HAZ [33].

Our results revealed an inverse association between PDI and the odds of being overweight and obese. Considering the vegetarian/ vegan diet, Desmond et al. found that a vegan (but not vegetarian) diet was associated with a lower BMI compared with an omnivore diet in Polish children aged 5–10 years old [29]. Furthermore, in a meta-analysis of observational studies, greater adherence to a vegan or vegetarian diet was associated with a lower BMI [34]. Most studies investigated the association between PDI and anthropometry conducted on adults. Among these studies, we can mention Satija et al. study, which showed that PDI was associated with less weight gain [24]. Also, investigators found an inverse association between PDI and BMI [35,36]. In another general population survey, investigators found a positive association between the frequency of animal-based food consumption and BMI [37]. However, conflicting evidence was observed in a systematic review of the vegetarian diet and children’s growth [20]. A cohort study found no significant association between high-starch plant-based dietary patterns and weight changes, which could be due to confounding factors in the study and non-differential misclassification of dietary intake [38].

The current study indicated an inverse association between hPDI scores and the risk of being underweight. A study showed that hPDI was associated with less weight gain [24]. In a systematic review and meta-analysis of observational studies, hPDI was inversely associated with weight and BMI [34]. A recent cross-sectional survey of children aged 6–9 found an inverse association between hPDI scores and abdominal obesity risk [39]. Another study in overweight and obese children ages 5–12 showed decreased BAZ after plant-based diet interventions [40]. Moreover, two meta-analyses indicated the positive effect of a plant-based diet on weight loss [41,42].

In line with previous studies, our study found that uPDI scores were positively associated with the risk of being overweight and obese. In a recent systematic review and meta-analysis in children and adolescents, higher intake of sugar-sweetened beverages, meat, and refined grains increased the odds of overweight/obesity [43]. Distinguishing overall, healthful and unhealthful plant-based dietary indices, an inverse association was found between PDI and hPDI with obesity risk, though uPDI was associated with higher obesity risk [44]. A recent systematic review of cohort studies suggested an inverse association between PDI and hPDI with body weight gain and a positive or null association between uPDI with weight gain [45]. In Satija et al. study, uPDI was associated with more weight gain [24]. However, in a cohort study of South Asian men and women, Bhupathiraju et al. found no association between uPDI with weight and BMI [35]. Furthermore, a study on Iranian pregnant women found no association between uPDI and inadequate or excessive weight gain [46]. The prospective design of these studies as well as the population of the studies, which were adults, could have caused a different result from our study.

We can explain the protective effect of plant-based foods by several possible mechanisms. First of all, plant foods are rich in fiber with a low energy density [46]. Fibers decrease macronutrient absorption, causing an increase in the levels of glucagon-like peptide-1 (GLP-1), cholecystokinin (CCK), and glucose-dependent insulinotropic polypeptide (GIP), inducing satiety [45,47]. Also, fiber ferments in the colon and changes the gut microflora, affecting energy balance by affecting how efficiently calories taken from the diet are used up [48]. Furthermore, they have anti-inflammatory properties and reduced inflammatory markers that can increase the risk of obesity [49,50]. On the other hand, plant-based foods are high in polyphenols, increasing thermogenesis and energy expenditure [51].

Our study has several strengths to be mentioned. The main strength is that this is the first study investigating the relationship between plant-based dietary patterns and anthropometry indices in primary school girls from Kerman City. Another strength is that we classified the plan-based dietary pattern as overall, healthful, and unhealthful plant-based dietary patterns. We designed a dish-based FFQ containing traditional Kerman foods. Furthermore, the effect of several possible confounders was adjusted in this study.

Some limitations of this study have to be discussed as well. The central limit is the study’s cross-sectional design, which would not allow us to find a cause-and-effect association between plant-based dietary patterns and anthropometry indices. Also, using FFQ for dietary assessment may lead to the misclassification of the participants.

## Conclusion

We concluded that adherence to plant-based dietary patterns lowers the risk of being overweight and obese. Consuming more unhealthy plant food increases the odds of being overweight and obese as well as has a negative outcome on height. Further investigations using large samples are needed to evaluate the effect of a plant-based diet on child growth, particularly with a prospective design.

## Supporting information

S1 Dataset Data files. These data are available in SPSS format (SAV) and is provided as a ZIP file containing the data files.
(RAR)
